# 3-(2-Amino-1,3-thia­zol-4-yl)-6-bromo-2*H*-chromen-2-one

**DOI:** 10.1107/S1600536809046674

**Published:** 2009-11-11

**Authors:** Deepak Chopra, A. R. Choudhury, K. N. Venugopala, Thavendran Govender, Hendrik G. Kruger, Glenn E. M. Maguire, T. N. Guru Row

**Affiliations:** a Department of Chemistry, Indian Institute of Science Education and Research, Bhopal 462 023, India; b Chemistry Group, Birla Institute of Technology and Science, Pilani, 333 031, Rajasthan, India; c School of Chemistry, University of Kwazulu-Natal, Durban 4000, South Africa; d School of Pharmacy and Pharmacology, University of Kwazulu-Natal, Durban 4000, South Africa; e Solid State and Structural Chemistry Unit, Indian Institute of Science, Bangalore 560 012, Karnataka, India

## Abstract

The mol­ecule of the title compound, C_12_H_7_BrN_2_O_2_S, is essentially planar with a maximum deviation of 0.234 (3) Å from the mean plane through all non-H atoms. The dihedral angle between the coumarin ring plane and that of the five-membered thia­zole ring is 12.9 (1)°. In the crystal, strong N—H⋯O, N—H⋯N and weak but highly directional C—H⋯O hydrogen bonds provide the links between the mol­ecules. In addition, C—H⋯π and π–π inter­actions [centroid–centroid distances = 3.950 (3)–4.024 (3) Å] provide additional stability to the inter­layer regions in the lattice.

## Related literature

For applications of coumarin compounds in photochemistry, see: Vishnumurthy *et al.* (2001 ▶). For their roles as dyes or laser dyes, see: Hooper *et al.* (1982 ▶); Nemkovich *et al.* (1997 ▶). For graph-set motifs, see: Bernstein *et al.* (1995 ▶). For the synthesis of the title compound, see: Venugopala *et al.* (2004 ▶). For related structures see: Vishnumurthy *et al.* (2001 ▶).
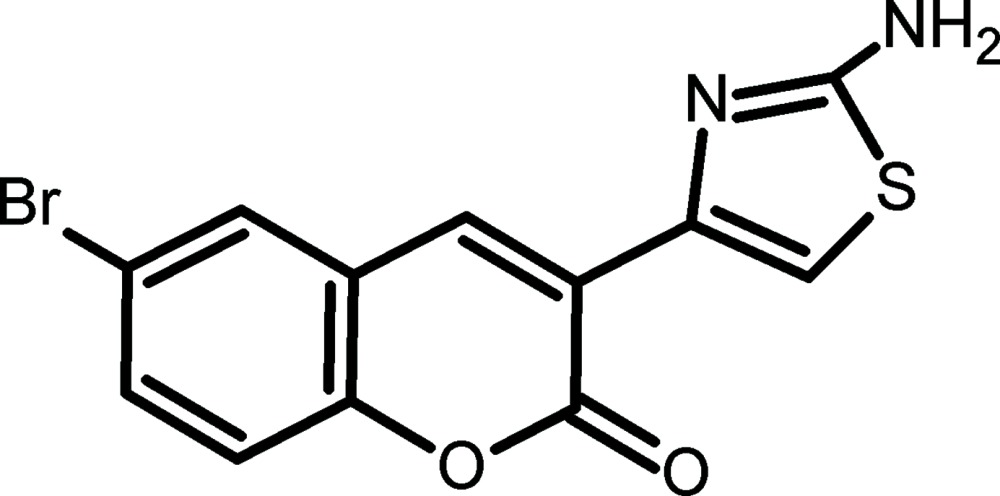


## Experimental

### 

#### Crystal data

C_12_H_7_BrN_2_O_2_S
*M* *_r_* = 323.17
Monoclinic, 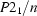

*a* = 7.031 (4) Å
*b* = 13.804 (8) Å
*c* = 12.453 (7) Å
β = 90.047 (9)°
*V* = 1208.6 (12) Å^3^
*Z* = 4
Mo *K*α radiation
μ = 3.57 mm^−1^
*T* = 290 K
0.32 × 0.12 × 0.11 mm

#### Data collection

Bruker SMART CCD area-detector diffractometer
Absorption correction: multi-scan (*SADABS*; Sheldrick,1996 ▶) *T*_min_ = 0.641, *T*_max_ = 0.675
9232 measured reflections
2431 independent reflections
2017 reflections with *I* > 2σ(*I*)
*R*_int_ = 0.019

#### Refinement

*R*[*F* ^2^ > 2σ(*F* ^2^)] = 0.034
*wR*(*F* ^2^) = 0.090
*S* = 1.03
2431 reflections
163 parameters
H-atom parameters constrained
Δρ_max_ = 0.54 e Å^−3^
Δρ_min_ = −0.56 e Å^−3^

### 

Data collection: *SMART* (Bruker, 2000 ▶); cell refinement: *SAINT* (Bruker, 2000 ▶); data reduction: *SAINT*; program(s) used to solve structure: *SHELXS97* (Sheldrick, 2008 ▶); program(s) used to refine structure: *SHELXL97* (Sheldrick, 2008 ▶); molecular graphics: *ORTEP-3 for Windows* (Farrugia, 1997 ▶) and *CAMERON* (Watkin *et al.*, 1993 ▶); software used to prepare material for publication: *PLATON* (Spek, 2009 ▶).

## Supplementary Material

Crystal structure: contains datablocks global, I. DOI: 10.1107/S1600536809046674/sj2669sup1.cif

Structure factors: contains datablocks I. DOI: 10.1107/S1600536809046674/sj2669Isup2.hkl

Additional supplementary materials:  crystallographic information; 3D view; checkCIF report

## Figures and Tables

**Table 1:** Hydrogen-bond geometry (Å, °)

*D*—H⋯*A*	*D*—H	H⋯*A*	*D*⋯*A*	*D*—H⋯*A*
N2—H2*A*⋯N1^i^	0.86	2.32	3.141 (4)	160
N2—H2*B*⋯O1^ii^	0.86	2.47	3.058 (3)	127
C4—H4⋯O1^iii^	0.93	2.38	3.304 (4)	172
C7—H7⋯*Cg*1^iv^	0.93	2.74	3.587 (4)	151

Symmetry codes: (i) 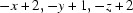; (ii) 
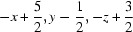
; (iii) 
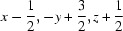
; (iv) 
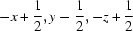
. *Cg*1 is the centroid of the thiazoyl ring.

**Fig. 1.**
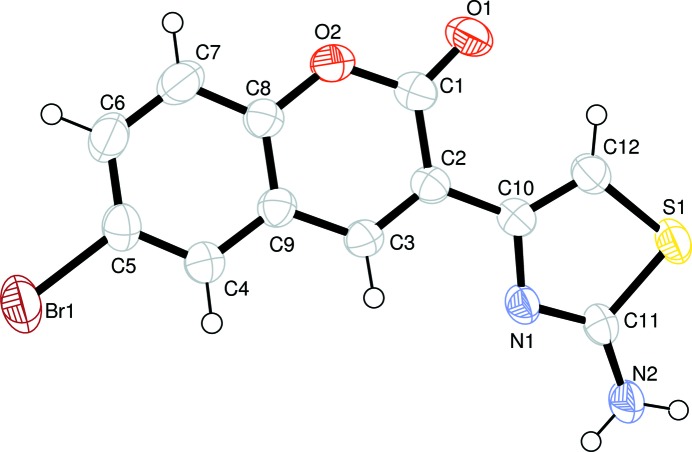
The structure of the title compound drawn with 50% probability displacement ellipsoids.

**Fig. 2.**
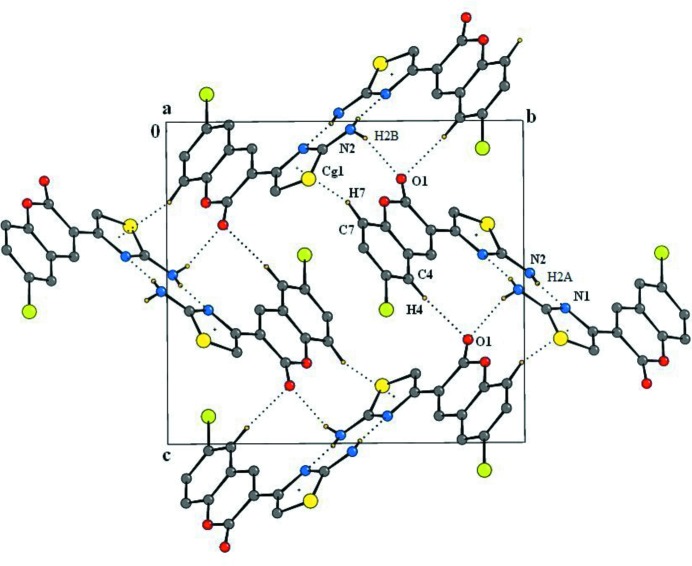
Packing diagram highlighting N—H···N/O hydrogen bonds and C—H···O intermolecular interactions. Only participating H atoms have been shown, others have been omitted for clarity.

## supplementary crystallographic information

### Comment

Coumarins are an important class of organic compounds and have been extensively studied. Such molecules of vast structural diversity find useful applications in several areas of synthetic chemistry, medicinal chemistry and photochemistry. The formation of [2 + 2] cycloaddition products upon irradiation (Vishnumurthy *et al.*,2001) of coumarin and its derivatives has contributed immensely to the area of solid-state chemistry. Several substituted coumarin derivatives find applications in the dye industry (Hooper *et al.*, 1982 )and in the area of laser dyes (Nemkovich *et al.*, 1997) based on the fact that such compounds show state dependent variations in their static dipole moments. The geometry and molecular packing patterns of several coumarins derivatives have been studied to evaluate the features of non-covalent interactions (Vishnumurthy *et al.*, 2001). Against this background, and to obtain more information on such compounds the solid-state structure of the title compound is reported here.

The molecular structure consists of a bromo substituted coumarin ring attached to an amino thiazoyl moiety (Figure 1). This compound crystallizes in a monoclinic centrosymmetric space group with *Z*'=1. The molecule is approximately planar, with a dihedral angle between the two rings being 12.9 (1) °. An analysis of the weighted least-squares plane through the coumarin ring C1/O2 and the thiazoyl ring shows that it is planar with the largest displacement of -0.019 (2)Å for C9. A characteristic Br···S short contact with distance 3.411 (2)Å is observed in the crystal lattice. Strong N—H···N and N—H···O hydrogen bonds (involving both H2A and H2B of the amino group with the ring nitrogen N1 and keto oxygen O1) form *R*^2^2(8) [Bernstein *et al.*, 1995] molecular dimers. These are linked by C(8) molecular chains along the crystallographic *b* axis forming a characteristic "chain of dimers". Furthermore, C—H···π interactions (involving H7 and the aromatic thiazoyl ring) provide additional stability forming chains along 'b' axis. Two such one-dimensional chains are linked by intermolecular C—H···O hydrogen bonds (involving H4 and O1) forming C(7) molecular chains along 'n' glide leading to the formation of a two dimensional sheet-like structure (Figure 2). π–π Stacking interactions involving the C4/C9 aromatic ring, Cg···Cg distance 3.950 (3)Å [Symmetry code: -*x* + 1, -*y*, -*z*] and between the thiazoyl ring and the C4/C9 ring [Cg···Cg distance = 4.024 (3) Å] [Symmetry code: *x* - 1, *y*, *z*] provide additional stability linking the layers of molecules.

### Experimental

The compounds were synthesized in accordance with the procedure reported in the literature (Venugopala *et al.*, 2004). Single crystals of the compound were grown both chloroform:methanol (1:1) by slow evaporation at 275–277 K.

### Refinement

All H-atoms were positioned geometrically and refined using a riding model with d(C-H) = 0.93Å, U_iso_=1.2U_eq_ (C) for aromatic and 0.86Å, U_iso_ = 1.2U_eq_ (N) for the NH atoms.

### Crystal data

**Table:**

C_12_H_7_BrN_2_O_2_S	*F*(000) = 640
*M**_r_* = 323.17	*D*_x_ = 1.776 Mg m^−^^3^
Monoclinic, *P*2_1_/*n*	Mo *K*α radiation, λ = 0.71073 Å
Hall symbol: -P 2yn	Cell parameters from 895 reflections
*a* = 7.031 (4) Å	θ = 1.5–25.8°
*b* = 13.804 (8) Å	µ = 3.57 mm^−^^1^
*c* = 12.453 (7) Å	*T* = 290 K
β = 90.047 (9)°	Needle, yellow
*V* = 1208.6 (12) Å^3^	0.32 × 0.12 × 0.11 mm
*Z* = 4	

### Data collection

**Table:**

Bruker SMART CCD area-detector diffractometer	2431 independent reflections
Radiation source: fine-focus sealed tube	2017 reflections with *I* > 2σ(*I*)
graphite	*R*_int_ = 0.019
φ and ω scans	θ_max_ = 26.4°, θ_min_ = 2.2°
Absorption correction: multi-scan (*SADABS*; Sheldrick,1996)	*h* = −8→8
*T*_min_ = 0.641, *T*_max_ = 0.675	*k* = −17→17
9232 measured reflections	*l* = −14→15

### Refinement

**Table:**

Refinement on *F*^2^	Primary atom site location: structure-invariant direct methods
Least-squares matrix: full	Secondary atom site location: difference Fourier map
*R*[*F*^2^ > 2σ(*F*^2^)] = 0.034	Hydrogen site location: inferred from neighbouring sites
*wR*(*F*^2^) = 0.090	H-atom parameters constrained
*S* = 1.03	*w* = 1/[σ^2^(*F*_o_^2^) + (0.0453*P*)^2^ + 0.6906*P*] where *P* = (*F*_o_^2^ + 2*F*_c_^2^)/3
2431 reflections	(Δ/σ)_max_ = 0.001
163 parameters	Δρ_max_ = 0.54 e Å^−^^3^
0 restraints	Δρ_min_ = −0.56 e Å^−^^3^

### Special details

**Table:**

Geometry. All e.s.d.'s (except the e.s.d. in the dihedral angle between two l.s. planes) are estimated using the full covariance matrix. The cell e.s.d.'s are taken into account individually in the estimation of e.s.d.'s in distances, angles and torsion angles; correlations between e.s.d.'s in cell parameters are only used when they are defined by crystal symmetry. An approximate (isotropic) treatment of cell e.s.d.'s is used for estimating e.s.d.'s involving l.s. planes.
Refinement. Refinement of *F*^2^ against ALL reflections. The weighted *R*-factor *wR* and goodness of fit *S* are based on *F*^2^, conventional *R*-factors *R* are based on *F*, with *F* set to zero for negative *F*^2^. The threshold expression of *F*^2^ > σ(*F*^2^) is used only for calculating *R*-factors(gt) *etc*. and is not relevant to the choice of reflections for refinement. *R*-factors based on *F*^2^ are statistically about twice as large as those based on *F*, and *R*- factors based on ALL data will be even larger.

### Fractional atomic coordinates and isotropic or equivalent isotropic displacement parameters (Å^2^)

**Table:**

	*x*	*y*	*z*	*U*_iso_*/*U*_eq_	
Br1	0.15344 (4)	0.88591 (3)	1.08569 (3)	0.06615 (15)	
S1	1.40939 (9)	0.60168 (5)	0.82189 (6)	0.04711 (19)	
N1	1.0835 (3)	0.61538 (14)	0.91596 (17)	0.0384 (5)	
N2	1.2684 (4)	0.48369 (18)	0.9743 (2)	0.0507 (6)	
O1	1.0382 (3)	0.84207 (14)	0.68021 (15)	0.0525 (5)	
O2	0.7679 (3)	0.88987 (13)	0.74985 (15)	0.0489 (5)	
C1	0.9241 (4)	0.83052 (17)	0.7517 (2)	0.0407 (6)	
C2	0.9369 (3)	0.75986 (16)	0.83941 (19)	0.0368 (5)	
C3	0.8015 (4)	0.75970 (18)	0.9159 (2)	0.0405 (6)	
C4	0.4977 (4)	0.82387 (19)	0.9903 (2)	0.0445 (6)	
C5	0.3471 (4)	0.88597 (19)	0.9794 (2)	0.0463 (6)	
C6	0.3340 (4)	0.9488 (2)	0.8935 (3)	0.0552 (7)	
C7	0.4756 (4)	0.9503 (2)	0.8172 (3)	0.0560 (7)	
C8	0.6280 (4)	0.88711 (17)	0.8272 (2)	0.0412 (6)	
C9	0.6421 (3)	0.82394 (17)	0.91312 (19)	0.0383 (5)	
C10	1.0974 (3)	0.69189 (17)	0.84259 (19)	0.0373 (5)	
C11	1.2368 (3)	0.56253 (17)	0.9130 (2)	0.0382 (5)	
C12	1.2599 (4)	0.6948 (2)	0.7847 (2)	0.0445 (6)	
H2A	1.1820	0.4644	1.0183	0.060*	
H2B	1.3722	0.4518	0.9676	0.060*	
H3	0.8123	0.7161	0.9728	0.048*	
H4	0.5037	0.7819	1.0485	0.053*	
H6	0.2303	0.9902	0.8875	0.066*	
H7	0.4694	0.9929	0.7595	0.068*	
H12	1.2876	0.7407	0.7324	0.053*	

### Atomic displacement parameters (Å^2^)

**Table:**

	*U*^11^	*U*^22^	*U*^33^	*U*^12^	*U*^13^	*U*^23^
Br1	0.0496 (2)	0.0818 (3)	0.0671 (2)	0.01305 (15)	0.01552 (15)	−0.00836 (16)
S1	0.0350 (3)	0.0504 (4)	0.0559 (4)	0.0006 (3)	0.0136 (3)	−0.0006 (3)
N1	0.0351 (10)	0.0393 (11)	0.0408 (11)	0.0034 (8)	0.0086 (9)	−0.0015 (9)
N2	0.0430 (13)	0.0463 (13)	0.0628 (16)	0.0119 (11)	0.0172 (12)	0.0071 (12)
O1	0.0600 (12)	0.0502 (11)	0.0474 (11)	−0.0031 (9)	0.0160 (9)	0.0069 (9)
O2	0.0570 (11)	0.0453 (10)	0.0445 (10)	0.0075 (8)	0.0061 (9)	0.0110 (8)
C1	0.0488 (14)	0.0358 (12)	0.0374 (13)	−0.0036 (11)	0.0051 (11)	−0.0033 (10)
C2	0.0408 (13)	0.0321 (11)	0.0374 (12)	−0.0021 (10)	0.0029 (10)	−0.0008 (10)
C3	0.0445 (14)	0.0371 (13)	0.0399 (14)	0.0044 (10)	0.0036 (11)	0.0048 (11)
C4	0.0452 (14)	0.0455 (14)	0.0429 (14)	0.0055 (11)	0.0039 (11)	0.0011 (12)
C5	0.0425 (14)	0.0498 (15)	0.0466 (15)	0.0068 (11)	0.0029 (12)	−0.0077 (12)
C6	0.0507 (16)	0.0538 (17)	0.0610 (18)	0.0174 (14)	−0.0025 (14)	0.0001 (14)
C7	0.0622 (18)	0.0512 (17)	0.0547 (17)	0.0134 (14)	−0.0053 (14)	0.0085 (14)
C8	0.0441 (14)	0.0401 (13)	0.0396 (14)	0.0026 (11)	0.0024 (11)	0.0013 (10)
C9	0.0409 (13)	0.0354 (12)	0.0386 (13)	0.0031 (10)	0.0011 (10)	−0.0009 (10)
C10	0.0396 (12)	0.0347 (12)	0.0376 (12)	−0.0029 (10)	0.0045 (10)	−0.0026 (10)
C11	0.0344 (12)	0.0380 (12)	0.0423 (13)	0.0000 (10)	0.0064 (10)	−0.0054 (10)
C12	0.0402 (14)	0.0444 (14)	0.0489 (15)	−0.0025 (11)	0.0083 (12)	0.0036 (12)

### Geometric parameters (Å, °)

**Table:**

Br1—C5	1.900 (3)	C3—C9	1.431 (4)
S1—C12	1.722 (3)	C3—H3	0.9300
S1—C11	1.748 (3)	C11—N2	1.344 (3)
N1—C11	1.303 (3)	C5—C6	1.379 (4)
N1—C10	1.399 (3)	C2—C1	1.469 (3)
O2—C1	1.370 (3)	C8—C7	1.383 (4)
O2—C8	1.379 (3)	C8—C9	1.384 (4)
C4—C5	1.369 (4)	C6—C7	1.380 (4)
C4—C9	1.399 (4)	C6—H6	0.9300
C4—H4	0.9300	C7—H7	0.9300
O1—C1	1.209 (3)	N2—H2A	0.8600
C10—C12	1.353 (4)	N2—H2B	0.8600
C10—C2	1.468 (3)	C12—H12	0.9300
C3—C2	1.348 (3)		
			
C12—S1—C11	88.97 (13)	O2—C8—C7	118.2 (2)
C11—N1—C10	110.3 (2)	O2—C8—C9	120.4 (2)
C1—O2—C8	123.0 (2)	C7—C8—C9	121.4 (3)
C5—C4—C9	119.6 (3)	O1—C1—O2	116.3 (2)
C5—C4—H4	120.2	O1—C1—C2	126.5 (2)
C9—C4—H4	120.2	O2—C1—C2	117.3 (2)
C12—C10—N1	115.4 (2)	C5—C6—C7	119.7 (3)
C12—C10—C2	128.1 (2)	C5—C6—H6	120.2
N1—C10—C2	116.5 (2)	C7—C6—H6	120.2
C2—C3—C9	122.3 (2)	C6—C7—C8	119.2 (3)
C2—C3—H3	118.8	C6—C7—H7	120.4
C9—C3—H3	118.8	C8—C7—H7	120.4
N1—C11—N2	124.9 (2)	C8—C9—C4	118.6 (2)
N1—C11—S1	114.80 (19)	C8—C9—C3	117.8 (2)
N2—C11—S1	120.32 (19)	C4—C9—C3	123.5 (2)
C4—C5—C6	121.4 (3)	C11—N2—H2A	120.0
C4—C5—Br1	119.0 (2)	C11—N2—H2B	120.0
C6—C5—Br1	119.5 (2)	H2A—N2—H2B	120.0
C3—C2—C10	121.5 (2)	C10—C12—S1	110.5 (2)
C3—C2—C1	119.1 (2)	C10—C12—H12	124.7
C10—C2—C1	119.4 (2)	S1—C12—H12	124.7

### Hydrogen-bond geometry (Å, °)

**Table:**

*D*—H···*A*	*D*—H	H···*A*	*D*···*A*	*D*—H···*A*
N2—H2A···N1^i^	0.86	2.32	3.141 (4)	160
N2—H2B···O1^ii^	0.86	2.47	3.058 (3)	127
C4—H4···O1^iii^	0.93	2.38	3.304 (4)	172
C7—H7···Cg1^iv^	0.93	2.74	3.587 (4)	151

Symmetry codes: (i) −*x*+2, −*y*+1, −*z*+2; (ii) −*x*+5/2, *y*−1/2, −*z*+3/2; (iii) *x*−1/2, −*y*+3/2, *z*+1/2; (iv) −*x*+1/2, *y*−1/2, −*z*+1/2.
